# High Energy Double Peak Pulse Laser Induced Plasma Spectroscopy for Metal Characterization Using a Passively Q-Switched Laser Source and CCD Detector

**DOI:** 10.3390/s19173634

**Published:** 2019-08-21

**Authors:** Juri Agresti, Andrea Azelio Mencaglia, Salvatore Siano

**Affiliations:** Istituto di Fisica Applicata “Nello Carrara”—CNR, 50019 Sesto Fiorentino, Italy

**Keywords:** double-pulse LIBS, copper alloys, calibration curves, arsenic, antimony, Egyptian bronze

## Abstract

Here, the development and testing of a portable double peak pulse laser induced plasma spectroscopy (DPP-LIPS) based on passively Q-switched Nd:YAG (Neodymium-doped Yttrium Aluminum Garnet) laser excitation is reported. The latter delivered structured laser pulses at a repetition rate of up to 20 Hz, including two energy peaks of about 100 mJ each with a relative temporal spacing of about 80 µs. Plasma spectra were collected using a low-cost Czerny–Turner spectrometer equipped with a non-intensified CCD (Charge-Coupled Device) array. Such a DPP-LIPS setup is technologically simpler and cheaper than the usual ones. Despite the relatively large temporal separation between the mentioned laser peaks, significant spectral intensity enhancements with respect to the usual single peak pulse configuration were observed. The amplification factor measured ranged between 2 and 10, depending on the specific emission peaks and the Q-switched configuration, and a consequent significant improvement of the detection limit of trace elements was observed. The instrument was calibrated for the quantitative analysis of copper alloy through systematic measurements carried out on reference samples and was then tested in an example archaeometric characterization of a statuette from the Egyptian Museum of Florence.

## 1. Introduction

Recent improvements of laser induced plasma/breakdown spectroscopy (LIPS or LIBS) contributed to promote the use of this technique for metal and mineral characterizations in archaeometrical, industrial, and environmental applications [1,2,3,4,5]. In particular, the possibility to perform rapid and relatively low-cost elemental depth profiles of metal artefacts in situ favored a number of applications in archaeometallurgical studies of large copper alloy sculptures [6] and statuettes [7]. However, the available portable devices [8] have a lower sensitivity than laboratory instruments. To date, this has not allowed for performing in situ alloy composition analysis extended to low-concentration and trace elements. Past studies and developments aimed at increasing the sensitivity of the technique have led to the introduction of a so-called double-pulse approach (DP-LIPS) in which a large enhancement of the signal-to-noise ratio has been observed [9,10,11,12,13,14]. This is based on the use of two laser pulses (both of which are delivered to the target along the same optical axis or along crossed directions), separated by a given time delay in the range of a few up to dozens of microseconds. The corresponding enhancement in the intensity of the spectral lines in the collinear configuration has been mostly attributed to a larger mass removal by the second pulse, which is favored by the rarefied atmosphere left by the expanding shock wave produced by the first pulse [15]. The signal increases documented with DP laser irradiation ranges from a factor of 2 up to orders of magnitude, depending on the experimental conditions (laser wavelength and intensity, delay between pulses, collection optics, etc.) and the energies of the atomic levels excited. This enhancing effect allows for improving the detection limits and hence extending the analysis to trace elements. However, most of the DP-LIPS systems developed so far employed two independent laser sources or a single-laser cavity suitably modulated using electro-optic components [16], along with the typical echelle spectrometers coupled to intensified CCD detectors. Such complex and expensive setups are not suitable for the implementation and widespread application of compact DP-LIPS.

In this work, a different approach is proposed using a passively Q-switched (QS) Nd:YAG (Neodymium-doped Yttrium Aluminum Garnet, laser wavelength of 1064 nm) laser source and compact spectrometers equipped with non-intensified CCD detectors. The passive Q-switching using a saturable absorber was preferred since it provides a relatively simple, inexpensive, and robust way to achieve multiple-peak QS laser emission with respect to the active methods based on Pockels cell and high voltage driver. In passive QS, when the gain and the total losses, including those of the saturable absorber itself, are equal, oscillations develop, which saturate the absorption of the passive component; then, the gain rises to the lasing level. With the appropriate selection of pumping and intra-cavity losses, it is possible to achieve a structured laser pulse including two or more QS peaks within the same pumping pulse. These consecutive peaks are temporally spaced by some tens of microseconds and individually have the same temporal shape and duration as that of a single peak QS laser pulse, which can be exploited to perform double peak pulse laser induced plasma spectroscopy (DPP-LIPS).

The main drawbacks of passive Q-switching are the temporal jitter and amplitude fluctuations induced by the laser dynamics, which could introduce significant fluctuations in the plasma emission of the second laser peak and associated plasma emission. Moreover, the mentioned temporal spacing of tens of microseconds could be too much with respect to the fluid-dynamics time evolution associated with the first laser peak. This work demonstrates that both these potential limitations can be effectively addressed using energy peaks around 100 mJ, which are relatively higher than the typical ones used in LIPS analysis. Furthermore, the water-cooled laser source used allowed a maximum pulse repetition frequency (prf) of 20 Hz, whereas previous DPP-LIPS reported in literature used air-cooled QS lasers emitting a few mJ at very low prf (maximum of 1 Hz) [8]. Although the latter may be enough for single shot applications, it is not practicable for more demanding depth profile applications of the technique, where up to some thousands of spectra are collected at each measurement site.

The system has been calibrated for the quantitative analysis of Sn, Pb, Zn, Ni, Fe, Sb, and As in copper alloys and has been successfully applied toward the characterization of an ancient bronze artefact from the Egyptian Museum of Florence. The comparison with the results of the analysis performed on the same artefact with the usual single peak pulse laser induced plasma spectroscopy (SPP-LIPS) clearly evidenced the improved analytical capabilities of the DPP setup in terms of detection limits for the mentioned elements.

## 2. Materials and Methods

### 2.1. LIPS Set-Up

**Figure 1:**
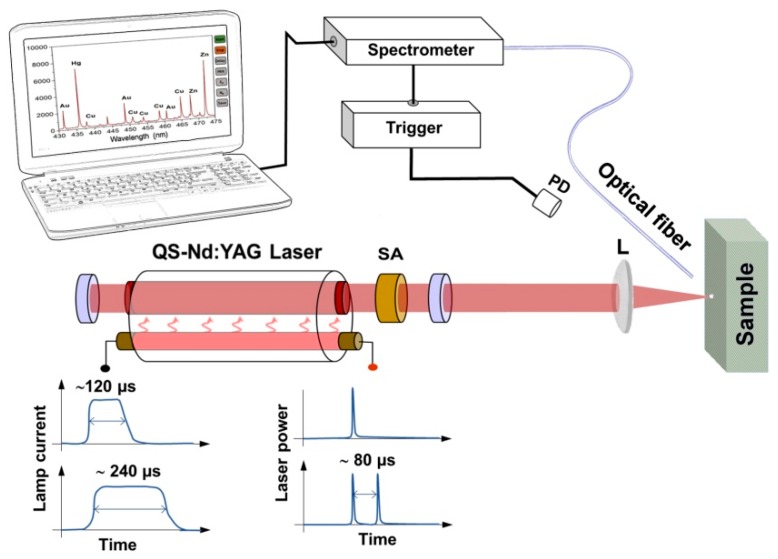
Schematics of the set-up. SA—saturable absorber, L—lens, PD—photodiode. Two operative condition are shown in the bottom part of the figure. Depending on the flashlamp pumping condition, it was possible to obtain a single Q-switched pulse or a couple of Q-switched pulses separated by about 80 µs.

A schematic of the set-up is given in Figure 1. The implemented laser system was composed of a nearly plane-parallel (10 m is the radius of curvature of the rear mirror) resonator 50 cm long, which consisted of a 6 × 85 mm Nd:YAG laser rod (with anti-reflection coated faces for 1.06 μm) housed in a pumping (elliptic cylinder-pump light concentrator) chamber with a linear xenon flashlamp, a 100% reflectivity rear mirror, and an output coupler with about 24% reflectivity at 1064 nm. The flashlamp power supply and pulse-forming electronics, as well as the cooling system of the pumping chamber, where adapted from an available commercial laser system. The system provided two possible settings of flashlamp discharge pulse length of approximately 120 µs and 240 µs. The water-based cooling system allowed for operating the laser at a repetition rate up to 20 Hz with an output energy, in the free running regime, of 500 ± 10 mJ with the longest pumping time. QS operation was achieved by inserting in the optical cavity a Cr^4+^: YAG saturable absorber [17] with a thickness of 4.3 mm, initial (unsaturated) transmission of 37% at the laser wavelength, and antireflection coatings on both faces. Laser pulses were monitored using a fast photodetector with a rise time of 600 ps and captured by a digital oscilloscope (LeCroy Wavesurfer 454, 500 MHz bandwidth). The laser output was measured using an energy meter. The laser beam was focused onto the sample surface in the perpendicular direction via a quartz plano-convex lens with a 75 mm focal length. The focus was set 1 mm beneath the irradiated surface in order to improve the reproducibility. The emitted light from the plasma plume was collected via a silica-silica optical fiber bundle (2 m long, 200 µm diameter, and 0.22 numerical aperture), positioned at 1 cm from the target surface and inclined at 40° with respect to the laser beam axis. The large acceptance angle of the fiber optic (≈25°) ensured that light from the entire plasma plume was collected. The fibers were connected to a set of commercial (Avantes, Apeldoorn, The Netherlands) Czerny–Turner compact spectrometers coupled to linear CCD detectors (2048 pixels). The complete set of six modules allowed for covering the spectral range of 200–890 nm with a maximum resolution better than 0.1 nm. In particular, in the following, we will refer to three wavelength ranges, 195–314 nm, 305–400 nm, and 405–500 nm, covered by the following configuration: 2400 lines/mm grating, 25 µm entrance slit, and detector dollection lens (DCL-UV/VIS) to enhance the sensitivity. The utilization of passive QS does not allow for precise triggering of both the laser emission and spectrum collection as is usual for an active QS laser [18]. Therefore, an optical trigger module based on a fast photodiode and suitable electronics was developed and provided the trigger signal to the spectrometer in correspondence with the desired QS laser peak. This allowed for easy selection between SPP and DPP LIPS modes. The acquisition of the DPP LIPS spectrum was triggered by the second QS peak. The intrinsic reset time of 1.3 µs of the sensor arrays used determines the minimum delay for acquisition of the LIPS signal. The integration time was set to the minimum available by our spectrometers, which was 2 ms, providing a time-integrated measurement of the plasma lifetime. The plasma emission spectra were collected in air at atmospheric pressure. The given geometry and timing of acquisition produced spatially and time-integrated LIPS measurements, which were expected to be less sensitive to plasma fluctuation.

### 2.2. Calibration

Nine reference bronze samples, whose composition is shown in Table 1, have been used in the calibration procedure. The surfaces of the samples were finely polished and cleaned. All the measurements were performed at a prf of 5 Hz by averaging 20 acquired spectra in order to improve the signal to noise ratio. For each bronze sample, a series of 400 DPPs were delivered to the target in order to average fluctuations due to sample in-homogeneity and possible depth-dependent effects [19], resulting in a series of 20 averaged spectra for each sample. Calibration was based on the measurement integrated intensities ratios. The emission lines selected for quantitative analysis were 296.12 nm and 458.7 nm for Cu, 284 nm for Sn, 283.3 nm for Pb, 472.22 nm for Zn, 275 nm for Fe, 300.3 nm for Ni, 231.15 nm for Sb, and 234.98 nm for As. After background subtraction, atomic line intensities were calculated using simple line integration for the Cu, Sn, Pb, Zn, Fe, and Ni lines, whereas a multi-peak fitting algorithm was used for the As and Sb lines in order to eliminate the effect of the interference with other lines. As per usual, the ratio between the integral of a selected peak of the element to be quantified and the integral of a reference line of the most abundant element (Cu) was calculated and the calibration curve was achieved by fitting the intensity ratios of interest as a function of the corresponding elemental weight fractions in the calibration sample. Limit of detection (LOD) for each calibrated element, was calculated as the concentration that produced a signal 3 times larger than the standard deviation of the signal associated with the blank sample (a sample not containing that element).

### 2.3. Test on Archaeological Bronze

**Figure 2:**
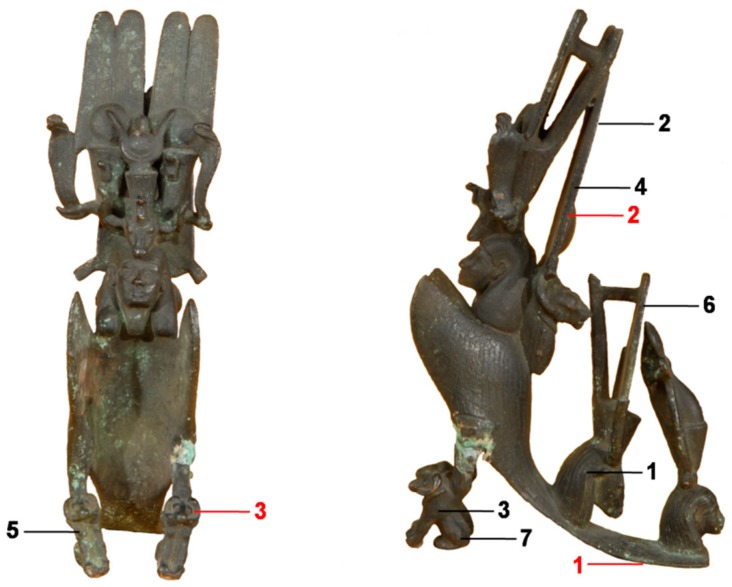
Front and side views of the bronze figurine (Inv. 5409) from the Egyptian Museum of Florence. LIPS measurement sites are also mapped with black and red labels, which refer to measurements performed using the DP-LIPS and SP-LIPS systems, respectively.

The calibrated DPP-LIPS system was validated in the archaeometallurgical study of the bronze statuette shown in Figure 2 from the Egyptian Museum of Florence (Inv. 5409, 8 × 14 × 25 cm). It represents a combination of Egyptian gods and was stylistically dated to the Late Period (664–332 BC). The main body represents a winged goddess (Isis) wearing an elaborate composite crown, the front pedestals represent two ram headed sphinxes, symbols of Amun, which is also sculpted in the back side, wearing the typical double-plumed Amun crown. Two other Egyptian gods are sculpted on the backside, wearing combinations of a Red Crown with the Atef Crown and with the double-plumed Amun crown. The left front-pedestal is attached to the main body by means of a mechanical join with evidences of previous restoration works. The surface of the object appears well-preserved and mostly covered with a homogeneous dark layer, likely attributable to a modern organic-matrix patination.

Seven quantitative elemental depth profiles were collected on the statuette, using 400 laser DPPs in each of the first three measurements sites and about 800 DPPs in the other four in order to characterize possible depth-dependent compositional variations induced by corrosion phenomena [20]. The corresponding alloy composition was calculated by averaging the measurements along the inner-most part of the respective depth profiles by discarding those measurements where exogenous elements, such as Ca were observed, whose presence in archaeological artefacts is due to the burial environment and weathering conditions. Moreover, three additional measurements were performed using the SP system described in Agresti et al. [18] in order to cross check the quantitative analysis of the elements for which both systems were calibrated and quantify the analytical improvement provided by the present set-up in trace analysis.

## 3. Results

**Figure 3:**
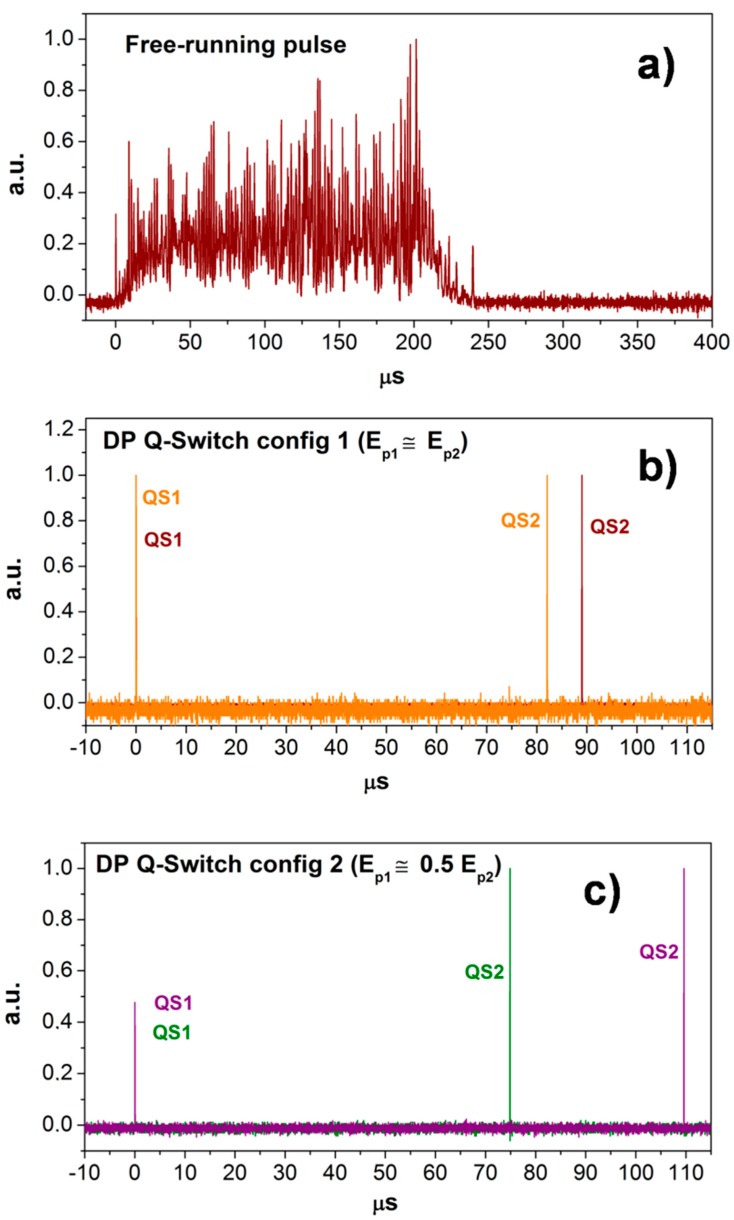
Examples of measured laser pulses in the long pumping regime (about 240 µs): (**a**) without a saturable absorber (free running), and with a saturable absorber in (**b**) configuration 1 and (**c**) configuration 2.

By changing the pumping duration of the flashlamp at constant voltage, it was possible to select between single- and double-peak QS-pulses (SPP and DPP). Using a flashlamp pulse of 120 µs, a single peak QS pulse of 90 ± 3 mJ (ε_p1_) and duration of about 20 ns was achieved. When the pumping pulse was set at 240 µs, two QS peaks of similar energy content were achieved in the laser pulse (total energy ε_p_ = ε_p1_ + ε_p2_ ≈ 2 ε_p1_ = 190 ± 3 mJ) with a temporal spacing of Δτ_p_ = 85 ± 5 µs (configuration 1). Starting from this condition, we found that a slight misalignment of the end-mirror allowed us to produce pulses, still including two QS peaks, but with different relative energies: ε_p1_/ε_p2_ = 0.3−0.5 (configuration 2). This operating regime exhibited slightly larger fluctuations in energy and temporal spacing (ε_p1_ + ε_p2_ = 200 ± 10 mJ, Δτ_p_ = 90 ± 20 µs), but, as shown in the following, it was very effective at producing LIPS signal intensification. Figure 3 shows the comparison among laser pulses as measured using the long pumping (240 µs) in the different operating conditions: (1) the free running, (2) well-aligned QS regime, and (3) slightly misaligned QS regime.

**Figure 4:**
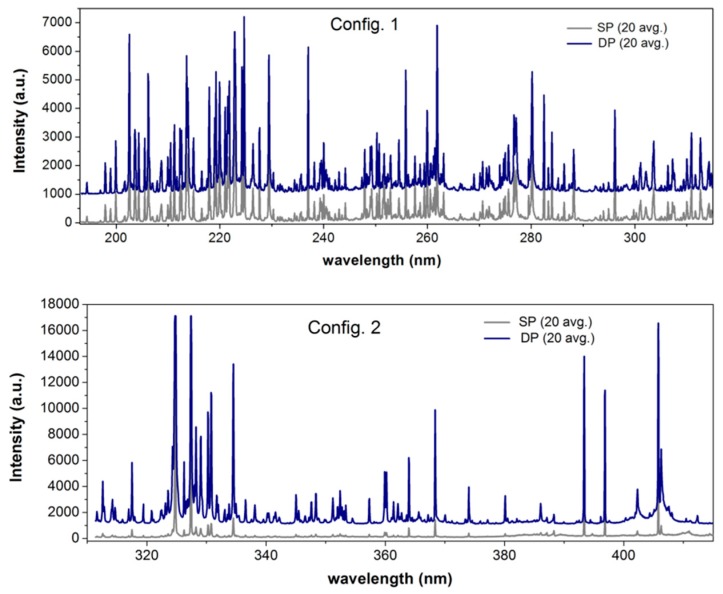
Comparison of SP and DP LIPS spectra of a bronze sample in the two studied configurations. DP spectra have been vertically shifted (1000 units) for better visualization. Acquisition delay of 1.3 µs, integration time of 2 ms, SP (ε_sp_ = 90 mJ), config. 1 DP (ε_p2_ = ε_p1_ = 85 mJ, Δτ_p_ = 85 µs), config. 2 DP (ε_p2_ = 2 ε_p1_ = 100 mJ, Δτ_p_ = 90 µs).

The three mentioned laser regimes, one SPP and two DPPs, were tested in the LIPS set-up described above without further optimization. Figure 4 shows the comparison between portions of the LIPS spectra acquired on a bronze sample in SPP mode, along with those collected using the two DPP settings described above: (a) ε_p2_ = ε_p1_, (b) ε_p2_ = 2 ε_p1_. The latter provided enhancements of about 2 and 10 times, respectively, over the SPP configuration, which agrees with previous studies on the dependence of the enhancement factor on the relative energy of the two consecutive excitations [21]. Thus, although the intrinsic Δτ_p_ and corresponding jitter of passive QS were relatively higher with respect to the typical ones used in literature, we found that they could still provide considerable improvements of the signal to noise ratio in DPP-LIPS in air when using high energy peaks. Moreover, as it is discussed in De Giacomo et al. [22], such high Δτ_p_ is of potential interest for underwater DPP-LIPS measurements. 

The most efficient configuration (ε_p2_ = 2 ε_p1_) was associated with a typical spectral line intensity fluctuation of about 30%, mirroring the mentioned jitter of the temporal spacing, Δτ_p_, whereas fluctuations using balanced peaks (ε_p2_ = ε_p1_) were limited to about 10% and therefore the latter condition was more reliable for applying the system in quantitative analyses. The DPP-LIPS set-up in configuration 1 was then selected as the reference configuration for calibration and following DPP measurements. 

**Figure 5:**
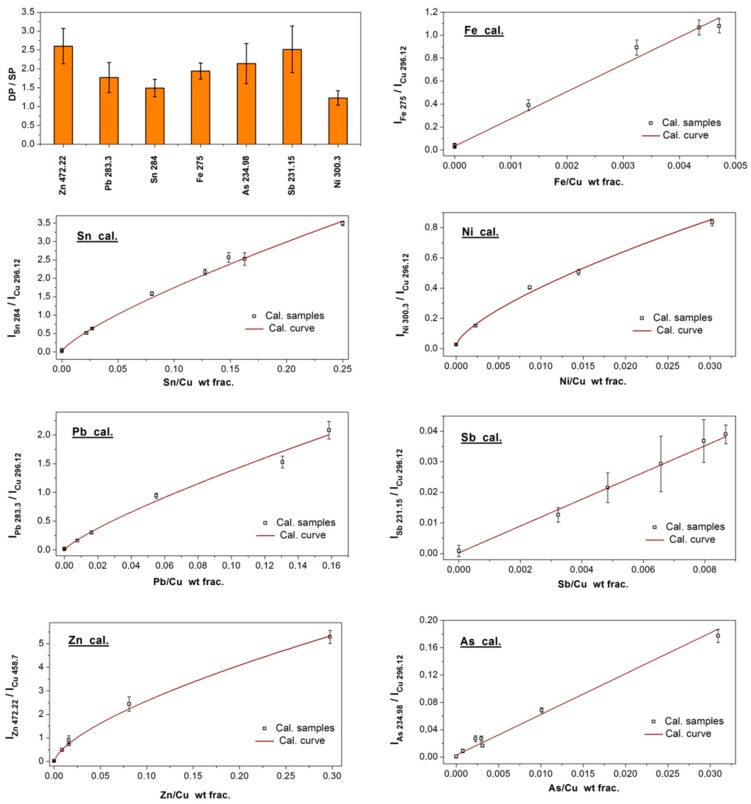
Top left: DPP enhancement factor in config. 1 for the atomic emission lines selected for calibration. The other panels show the calibration curves obtained in config. 1 for the quantification of Sn, Pb, Zn, Fe, Ni, Sb, and As in copper alloys. Sn: $y=0.012+10.4x^{0.78}$ (R^2^ = 0.99), Pb: $y=8.9x^{0.81}$ (R^2^ = 0.85), Zn: $y=12x^{0.67}$ (R^2^ = 0.98), Fe: y=0.035+237x (R^2^ = 0.98), Ni: $y=0.026+9.8x^{0.71}$ (R^2^ = 0.99), Sb: y=0.005+4.38x (R^2^ = 0.99), As: y=0.003+5.97x (R^2^ = 0.96). Acquisition delay of 1.3 µs, integration time of 2 ms, SP (ε_sp_ = 90 mJ), config. 1 DP (ε_p2_ = ε_p1_ = 85 mJ, Δτ_p_ = 85 µs).

The top-left of Figure 5 reports the enhancement factors of DPP (ε_p2_ = ε_p1_) versus SPP operations of the elemental lines selected for calibration, as calculated using the ratio of the corresponding integrated intensities. The observed enhancement in double-pulse LIPS could be roughly correlated with the upper energy level of the corresponding emission transition. This result was an indication that the intensity enhancement was at least partially due to an increase in the plasma temperature (due to the Boltzmann factor governing the population distribution). The remainder of Figure 5 presents all seven calibration curves obtained with the procedure explained above. The calibration curves were achieved through the fitting of the experimental data using suitable linear or power functions. The LOD (limit of detection), calculated via converting the 3σ signal (three times the standard deviation of the background-subtracted blank signal) to weight fractions using the corresponding calibration curves were: 70 ppm for Sn, 120 ppm for Pb, 13 ppm for Zn, 60 ppm for Fe, 53 ppm for Ni, 1400 ppm for Sb, and 1000 ppm for As. It is worth noting that an order of magnitude of improvement in the detection limits of the main alloying elements (Sn, Zn) and Pb with respect to the to the performances of the portable SPP-LIPS system we previously reported [18]. To our best knowledge, this is the first DPP-LIPS system, based on saturable absorber QS and non-intensified detector that has been calibrated and applied for the quantification of seven elements (Sn, Pb, Zn, Fe, Sb, As, and Ni) in copper alloys.

**Figure 6:**
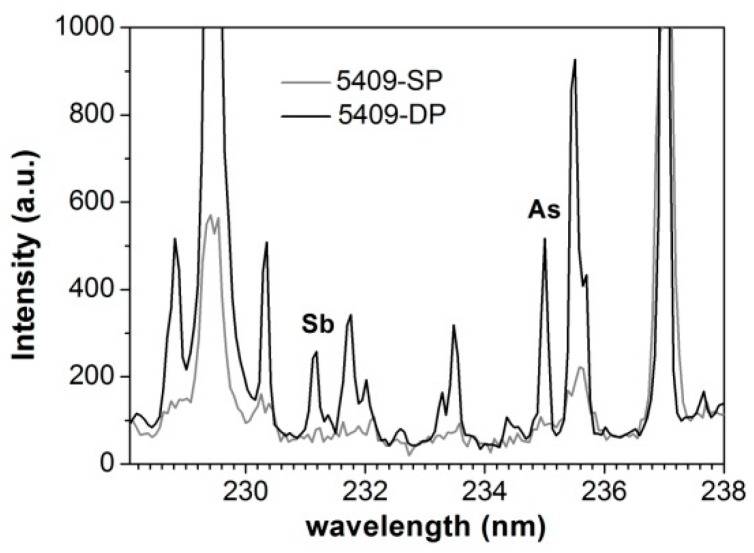
Comparison of two averaged (20 measurements) spectra acquired in close proximity on the Egyptian bronze figurine with the proposed DPP- and the SP-LIPS system of Agresti et al. [18].

The possibility to perform a reliable quantitative analysis of trace elements with the new set-up, in particular of As and Sb, was fully exploited in the study of the ancient bronze artifact displayed in Figure 2. In the following, the single-pulse (SP) LIPS system, with which we compared the results, is the portable system (calibrated for the analysis of copper alloys) described in Agresti et al. [18]. The comparison of a portion of the spectrum collected using the SP and DPP set-up, shown in Figure 6, clearly demonstrates the significant improvement achieved. In particular, DPP-LIPS provided a signal to noise ratio for the detection of As and Sb, which was much higher than that of the SP set-up. It is worth stressing that a thoughtful comparison of the two LIPS systems should also consider the differences in the optics for collecting the plasma emission and in the laser beam energies, but this is beyond the purpose of the present paper.

The results of seven quantitative measurements performed on the Egyptian statuette described above using the present DPP-LIPS system are listed in Table 2, along with three measurements carried out with the SPP system. These show the object was cast using a leaded bronze with a relatively high lead content (a common practice in Egyptian metallurgy during the Late Period [23,24]) and that the results provided by the two systems on the major elements were congruent. A good agreement was found also for the joined part (left pedestal: sites 3, 7, and SP_3), although the analyses revealed it is a low brass, a composition completely different with respect to the main alloy. Conversely, a high As content was measured using DPP-LIPS in the main alloy (about 2%) that was not detected using SPP-LIPS. Furthermore, DPP-LIPS allowed for achieving the trace element pattern, which gave very repeatable results among the various sites of the artefact analyzed. In particular, the main alloy included a relatively high content of Sb (about 0.5%), whereas neither Sb nor As were detected in the left pedestal whose brass was rather pure, thus supporting the conclusion it was likely added in a modern restoration.

In some more details, the comparison of the compositions measured in the sites 2 and 4 and the sites 3 and 7 indicates that the set of measurements performed at 400 DPP could still be affected by the near-surface compositional alteration induced by corrosion phenomena. As a matter of fact, measurements 4–7 showed a better agreement with the results of the measurements performed with the SPP portable LIPS, which were carried out using about 1500 single pulses at each measurement site.

## 4. Conclusions

A novel portable DPP-LIPS system was developed and successfully tested on reference metal samples and an archaeological copper alloy statuette. We demonstrated that the use of a passively QS Nd:YAG laser system, along with a non-intensified linear detector, provide an effective and reduced-cost alternative to the standard DP systems employing two synchronized lasers or modulated electro-optic switches. The achieved sensitivity allowed for measuring trace elements in copper alloys of interest in metallurgical studies within the archaeometric and industrial fields. Some of the key features of the present DPP-LIPS set-up are given by the relatively high energy per pulse and prf it is able to produce (about 100 mJ and 20 Hz, respectively), as well as the high delay between first and second laser peak (around 80 µs). These operative conditions are desirable whenever LIPS depth profiles and/or fast analysis are required and were here implemented for the first time in a portable DPP-LIPS system employing passive Q-switching. Further investigations are foreseen in order to develop a stable configuration for a reliable exploitation of the observed maximum DPP-LIPS signal enhancement of a factor of 10, as well as for exploiting the present DPP approach in underwater LIPS analyses where the present temporal separation between the energy peaks of the laser pulse are expected to further improve the detection limits.

## Figures and Tables

**Table 1:** Reference calibration samples for quantitative analysis (element concentration in weight percentage, wt%).

	Sn	Pb	Zn	Fe	Sb	As	Ni	Cu
CS1	1.63	0.58	22.13	0.35	0.36	0.17	0.17	74.36
CS2	9.45	11.74	1.17	0.24	0.59	0.22	2.24	74.08
CS3	11.3	9.94	0.66	0.1	0.5	0.06	1.1	74.11
CS4	6.46	4.43	6.52	0.35	0.26	0.25	0.7	80.48
CS5	2.5	1.5	1.5	--	0.8	--	--	93.7
CS6	14	--	--	--	--	--	--	86
CS7	20	--	--	--	--	--	--	80
CS8	--	--	--	--	--	1	--	99
CS9	--	--	--	--	--	3	--	97

**Table 2:** Results of LIPS measurements on Egyptian statuette (Inv. 5409). One to seven measurements were performed with the new DPP-LIPS. SP_1–3 measurements performed with the SPP-LIPS system described in Agresti et al. [18]. n.d. = not detected (because below LOD).

Site	Sn (wt%)	Pb (wt %)	Zn (wt %)	Fe (wt %)	Sb (wt%)	As (wt%)	Ni (wt %)
**1**(400DP)	5.5± 0.4	19.6 ± 1.2	0.0045 ± 0.0008	0.012 ± 0.002	0.55 ± 0.08.	2.1 ± 0.1	0.06 ± 0.02
**2**(400DP)	4.2 ± 0.2	28.9 ± 2.4	0.0062 ± 0.0016	0.007 ± 0.002	0.57 ± 0.02	1.8 ± 0.1	0.03 ± 0.01
**3**(400DP)	2.2 ± 0.2	1.5 ± 0.1	5.8 ± 0.4	0.086 ± 0.004	n.d.	n.d.	0.10 ± 0.01
**4**(800DP)	5.1 ± 0.2	20.6 ± 3.0	0.0058 ± 0.0011	0.010 ± 0.003	0.37 ± 0.05	2.2 ± 0.2	0.03 ± 0.01
**5**(800DP)	5.6 ± 0.5	21.5 ± 2.6	0.0046 ± 0.0007	0.003 ± 0.002	0.49 ± 0.06	1.9 ± 0.2	0.07 ± 0.01
**6**(800DP)	4.9 ± 0.2	14.3 ± 1.3	0.0063 ± 0.0009	0.007 ± 0.003	0.32 ± 0.06	2.0 ± 0.1	0.06 ± 0.01
**7**(800DP)	1.2 ± 0.1	0.7 ± 0.2	8.2 ± 0.8	0.043 ± 0.004	n.d.	n.d.	0.05 ± 0.01
**SP_1**	4.6 ± 0.3	17.2 ± 2.5	n.d.	n.d.	n.d.	n.d.	n.d.
**SP_2**	5.2 ± 0.7	21.7 ± 1.9	n.d.	n.d.	n.d.	n.d.	n.d.
**SP_3**	1.4 ± 0.3	1.1 ± 0.3	8.9 ± 0.9	n.d.	n.d.	n.d.	n.d.
